# High-speed quantitative optical imaging of absolute metabolism in the rat cortex

**DOI:** 10.1117/1.NPh.8.2.025001

**Published:** 2021-04-08

**Authors:** Robert H. Wilson, Christian Crouzet, Mohammad Torabzadeh, Afsheen Bazrafkan, Niki Maki, Bruce J. Tromberg, Yama Akbari, Bernard Choi

**Affiliations:** a University of California, Department of Medicine, Irvine, California, United States; b University of California, Beckman Laser Institute, Irvine, California, United States; c University of California, Health Policy Research Institute, Irvine, California, United States; d University of California, Department of Biomedical Engineering, Irvine, California, United States; e University of California, Department of Neurology, Irvine, California, United States; f University of California, School of Medicine, Irvine, California, United States; g University of California, Department of Surgery, Irvine, California, United States

**Keywords:** brain metabolism, cerebral metabolic rate of oxygen, cerebral blood flow, diffuse optical imaging, cardiac arrest, brain ischemia

## Abstract

**Significance:** Quantitative measures of blood flow and metabolism are essential for improved assessment of brain health and response to ischemic injury.

**Aim:** We demonstrate a multimodal technique for measuring the cerebral metabolic rate of oxygen (${\mathrm{CMRO}}_{2}$) in the rodent brain on an absolute scale ($\mu M\;O_{2}/\min$).

**Approach:** We use laser speckle imaging at 809 nm and spatial frequency domain imaging at 655, 730, and 850 nm to obtain spatiotemporal maps of cerebral blood flow, tissue absorption ($\mu_{a}$), and tissue scattering (${\mu_{s}}^{\prime }$). Knowledge of these three values enables calculation of a characteristic blood flow speed, which in turn is input to a mathematical model with a “zero-flow” boundary condition to calculate absolute ${\mathrm{CMRO}}_{2}$. We apply this method to a rat model of cardiac arrest (CA) and cardiopulmonary resuscitation. With this model, the zero-flow condition occurs during entry into CA.

**Results:** The ${\mathrm{CMRO}}_{2}$ values calculated with our method are in good agreement with those measured with magnetic resonance and positron emission tomography by other groups.

**Conclusions:** Our technique provides a quantitative metric of absolute cerebral metabolism that can potentially be used for comparison between animals and longitudinal monitoring of a single animal over multiple days. Though this report focuses on metabolism in a model of ischemia and reperfusion, this technique can potentially be applied to far broader types of acute brain injury and whole-body pathological occurrences.

## 1 Introduction

Assessing brain metabolism on a quantitative scale is critical for improved diagnosis, monitoring, and treatment of a wide variety of acute brain injury caused, for example, by ischemia, hemorrhage, and trauma. Indeed, numerous studies show abnormal brain metabolism in such acute brain injury in patients.^1^ Measurements of the cerebral metabolic rate of oxygen (${\mathrm{CMRO}}_{2}$) may provide insight into the viability of brain tissue following ischemia.^2^ In particular, knowledge of ${\mathrm{CMRO}}_{2}$ in absolute units would obviate the need for baseline measurements and facilitate longitudinal measurements to track longer-term cerebral recovery following ischemia and reperfusion. Indeed, in a clinical setting where patients present with an acute brain injury, there is often no way to obtain a baseline (preinjury) measurement, underscoring the need for translational research supporting absolute rather than relative measurements. Also, absolute ${\mathrm{CMRO}}_{2}$ measurements would enable quantitative comparisons between the values of different subjects at baseline and at subsequent time points in preclinical or clinical studies. These needs are currently unmet, as no existing technology directly and noninvasively measures absolute ${\mathrm{CMRO}}_{2}$ with both high spatial and temporal resolution using intrinsic signals.

Unfortunately, conventional clinical monitoring techniques (e.g., arterial blood pressure, pulse oximetry, laser Doppler flowmetry, brain oxygenation monitors, and jugular bulb oximetry) typically cannot separate alterations in cerebral metabolism from changes in blood flow. Established techniques to measure ${\mathrm{CMRO}}_{2}$ include medical imaging modalities such as positron emission tomography (PET) and functional magnetic resonance imaging (fMRI). PET^3^^–^^7^ can measure absolute ${\mathrm{CMRO}}_{2}$, but it is expensive and nonportable, and requires use of exogenous contrast agents containing radioactive tracers. fMRI measures ${\mathrm{CMRO}}_{2}$ changes via the blood oxygen level dependent (BOLD) signal, but it provides absolute ${\mathrm{CMRO}}_{2}$ only with extensive calibration, as the BOLD signal only serves as a surrogate for cerebral blood flow (CBF) and hemoglobin content and not as a direct measurement of these quantities. In addition, both PET and BOLD fMRI typically have limited temporal resolution and cannot be performed repeatedly on a patient over a short period to monitor hyperdynamic changes caused by acute insults.

Diffuse optical spectroscopy and diffuse optical imaging (DOI) techniques are an attractive alternative, as they are noncontact and use measurements of low-irradiance visible and near-infrared light to extract endogenous tissue absorption and scattering coefficients. Further analysis of the absorption coefficient yields measurements of relative changes in hemoglobin content and oxygen saturation.^8^ Frequency-modulated and time-resolved techniques^9^ enable absolute measurements of these two parameters. Coherent light techniques, such as diffuse correlation spectroscopy, enable measurements of blood flow.^10^ Combined use of these techniques can yield measurements of ${\mathrm{CMRO}}_{2}$. However, the majority of these techniques have limited spatial resolution, oftentimes serving as point measurements.

Recently, we demonstrated that the combination of spatial frequency domain imaging (SFDI) and laser speckle imaging (LSI) can quantify tissue metabolic changes with both high spatial and temporal resolution.^11^ We recently applied high-speed LSI^12^ and SFDI^8^ to measure perfusion, oxygenation, and tissue scattering in the brain in a preclinical cardiac arrest (CA) model of global cerebral ischemia that demonstrates a highly dynamic course of ischemia and reperfusion. The capability of our rapid multimodal SFDI + LSI system to image blood flow and hemoglobin concentration simultaneously enables high-speed measurement of ${\mathrm{CMRO}}_{2}$.^13^ Furthermore, we can account for the effects of time-varying tissue scattering at multiple wavelengths^14^ and the contribution of venous regions versus parenchyma when calculating ${\mathrm{CMRO}}_{2}$. Here, we report on our approach to analyze our multimodal optical imaging data with a mathematical model of ${\mathrm{CMRO}}_{2}$ that incorporates a “zero-flow” boundary condition during the ischemia phase of our CA model, to obtain the parameters necessary for absolute ${\mathrm{CMRO}}_{2}$ measurements.

## 2 Methods

### 2.1 Animal Preparation

The Institutional Animal Care and Use Committee (IACUC) at the University of California, Irvine (protocol number 2013-3098-01) approved all procedures described in this report. Ten male Wistar rats (weight ∼300 to 400 g) were imaged, and details of the animal preparation procedures are described in our previous publications.^8^^,^^12^^,^^15^^,^^16^ Before the experiment, all subjects were endotracheally intubated under isoflurane anesthesia. Each subject had epidural screw electrodes implanted for electrocorticography (ECoG) and a hemicraniectomy (4 mm right-to-left × 6 mm anterior-to-posterior) was performed to enable imaging of a portion of the right sensory and visual cortices. Cannulation of the femoral artery allowed the delivery of drugs, sampling of blood, and monitoring of blood pressure.

### 2.2 Cardiac Arrest and Cardiopulmonary Resuscitation

**Fig. 1:**
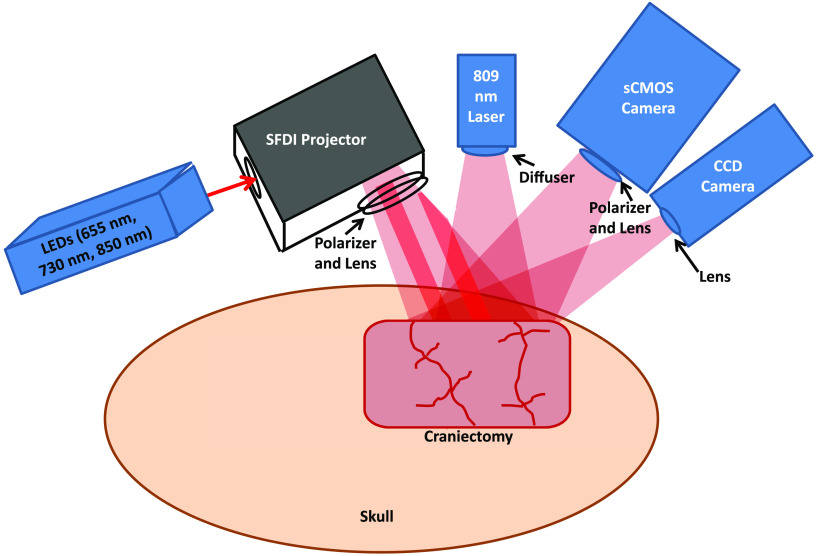
Multimodal platform (not to scale) for LSI and multispectral SFDI of the rat brain. A craniectomy (∼6  mm×4  mm area) is performed to provide direct access to the brain for optical imaging. For SFDI, LEDs of 655, 730, and 850 nm are sequentially sent into a spatial light modulator that acts as a projector to send spatially modulated patterns of light onto the brain.^8^ A scientific CMOS camera detects the backscattered light. For LSI, an 809-nm laser illuminates the brain with coherent light, and the remitted speckle pattern is captured at 60 fps with a CCD camera.

Figure 1 shows the multimodal setup employed in the experiments. At the onset of each experiment, the level of isoflurane was decreased from 2% to 0.5% to 1.0%. Concurrently, the mixture of inhaled gases was altered from $50\%\;O_{2}+50\%\;N_{2}$ to 100% $O_{2}$. Two minutes later, to reduce confounding effects of isoflurane on cerebral perfusion and metabolism,^17^ the anesthesia was turned off, at which time the subject breathed room air (21% $O_{2}$). During this same period, 1 mL of 2  mg/kg Vecuronium (a neuromuscular blocker) and 1 mL of heparinized saline were administered intravenously, which led to respiration controlled solely by the ventilator. This stage of the experiment lasted for 3 min, after which the ventilator was turned off to induce asphyxia, leading to progressive hypoxic hypercarbic hypotension. CA was defined as the period over which the pulse pressure was below 10 mmHg and systolic pressure below 30 mmHg. The conditions of these experiments induced pulseless electrical activity, which is common in CA patients in a hospital setting.

Forty-five seconds before the end of the CA period, the ventilator was turned on (respiratory rate=85  breaths/min, PIP=17.5 to $18.5\;\mathrm{cm}\;H_{2}O$, $\mathrm{PEEP}=3\;\mathrm{cm}\;H_{2}O$ at 2.5 LPM), and 100% oxygen was delivered. Immediately before the onset of cardiopulmonary resuscitation (CPR), 0.01  mg/kg epinephrine, 1  mmol/kg sodium bicarbonate, and 2 mL of heparinized saline were administered intravenously. Then, CPR was performed via external cardiac massage and terminated upon return of spontaneous circulation (ROSC), as identified from arterial blood pressure measurements. Subsequently, the animal was monitored continuously with arterial blood pressure, optical imaging, and ECoG for an additional ∼2  h, after which the animal was euthanized with pentobarbital. As a quantitative measurement of the information content contained in the ECoG signals, we calculated an entropy-based parameter known as ECoG information quantity (IQ).^18^ Recovery of ECoG signal following ROSC was quantified by (1) time to initial resumption (burst) of ECoG activity and (2) ECoG IQ 90 min post-ROSC (as in Ref. 19).

### 2.3 Laser Speckle Imaging

For LSI, an 809-nm laser with long coherence length (Ondax, Monrovia, California) served as the light source. To increase uniformity of illumination over the imaged region of interest (ROI), a ground-glass diffuser (ThorLabs, Inc., Newton, New Jersey) was placed between the laser and the brain. A CCD camera (Point Grey Research Inc., Richmond, BC, Canada) detected the backscattered light with a 10-ms exposure time, resulting in image acquisition at a frame rate of 60 Hz. Using a 5×5 sliding spatial window filter, the equation K=σ/⟨I⟩ was employed to calculate the local speckle contrast K at each pixel, where ⟨I⟩ is the mean intensity within the filter and σ is the standard deviation within the filter.^20^ Then, the speckle flow index (SFI) was determined from the values of K and the exposure time T via a simplified speckle imaging equation $\mathrm{SFI}=1/(2TK^{2})$.^20^ Time-resolved SFI curves were generated by taking the mean of the SFI over a selected ROI at each time point.

### 2.4 Spatial Frequency-Domain Imaging

For SFDI, light-emitting diodes (LEDs) of three different wavelengths (655, 73, and 850 nm) were used as light sources. The light was directed to a spatial light modulator that projected square-wave patterns onto the brain.^8^ Backscattered light was captured using a scientific complementary metal-oxide-semiconductor (sCMOS) camera (Hamamatsu Photonics). An Arduino Due microcontroller board was used to synchronize the camera acquisition, spatial light modulator, and LEDs. For each wavelength, four patterns were projected onto the tissue in sequence. The first pattern was nonmodulated (i.e., DC illumination), and the three subsequent patterns were modulated at spatial frequency $∼0.3\;{\mathrm{mm}}^{-1}$ with three distinct spatial phases to enable demodulation.^21^ Thus, there were a total of (3 wavelengths × 4 frames) = 12 frames of SFDI data for each measurement time point. The detected square wave pattern could be approximated as a sinusoid, allowing demodulation in the manner described previously by our group.^22^ With this acquisition scheme, we were able to reconstruct tissue hemodynamics and ${\mathrm{CMRO}}_{2}$, at an effective imaging rate of ∼14  Hz.

After demodulating the spatially modulated data, the diffuse reflectance at each time point and wavelength was calculated from the raw data via calibration against a tissue-simulating phantom with known optical properties.^21^ The diffuse reflectance maps were then fit with a Monte Carlo model to extract the tissue absorption coefficient $\mu_{a}$ and reduced scattering coefficient ${\mu_{s}}^{\prime }$ at each wavelength.^8^ Next, the average ${\mu_{s}}^{\prime }$ was determined for a selected ROI and a new $\mu_{a}$ determined using diffuse reflectance with the nonmodulated pattern and this average ${\mu_{s}}^{\prime }$. To calculate the concentrations of oxygenated and deoxygenated hemoglobin (${\mathrm{ctHbO}}_{2}$ and ctHb, respectively) within the tissue, this new $\mu_{a}(\lambda )$ spectrum was fit with the model spectrum $\mu_{a}(\lambda )=2.303({\mathrm{ctHbO}}_{2}\varepsilon_{\mathrm{HbO}2}+\mathrm{ctHb}\varepsilon_{\mathrm{Hb}})$, where $\varepsilon_{\mathrm{HbO}2}$ and $\varepsilon_{\mathrm{Hb}}$ were the molar extinction coefficients of oxy- and deoxyhemoglobin, respectively. The total tissue hemoglobin concentration (${\mathrm{ctHb}}_{\mathrm{tot}}$) was calculated by summing ctHb and ${\mathrm{ctHbO}}_{2}$. The tissue oxygen saturation was determined using the equation ${\mathrm{StO}}_{2}={\mathrm{ctHbO}}_{2}/({\mathrm{ctHbO}}_{2}+\mathrm{ctHb})$.

#### 2.4.1 Correction of speckle flow index for tissue absorption and scattering

K, and hence SFI, depends on local optical properties.^23^ To correct the measured SFI for dynamic optical properties, the measured K values were converted to a characteristic flow speed ($v_{c}$) by using the following equation:^23^

$$K^{2}=\frac{(\frac{2}{T})\int_{0}^{T}\beta G_{1}^{2}(\tau )(1-\tau /T)d\tau }{G_{1}^{2}(\tau =0)},$$ (1)

β is a constant (typically set to 1) related to polarization and coherence properties of the LSI instrumentation. From the Siegert relationship, the intensity autocorrelation function $G_{2}(\tau )$ is related to $G_{1}(\tau )$, which, in turn, is described by the correlation diffusion equation:

$$\nabla^{2}G_{1}(\tau )-\mu_{\mathrm{eff}}G_{1}(\tau )=q.$$ (2)

In Eq. (2), q is the source term; and $\mu_{\mathrm{eff}}={\left(3\mu_{a,\mathrm{dyn}}\mu_{\mathrm{tr}}\right)}^{1/2}$, where $\mu_{\mathrm{tr}}=(\mu_{a}+{\mu_{s}}^{\prime })$ is the tissue transport coefficient and $\mu_{a,\mathrm{dyn}}=(\mu_{a}+{\mu_{s}}^{\prime }k_{o}^{2}\langle \Delta r^{2}(\tau )\rangle /3)$ the dynamic tissue absorption coefficient.^23^ In the equation for $\mu_{a,\mathrm{dyn}}$, $\left\langle \Delta r^{2}(\tau )\right\rangle$ is the mean square displacement of the moving scatterers (i.e., the red blood cells) and $k_{o}$ is the photon wavenumber. Solving Eq. (2), $G_{1}(\tau )$ can be written as

$$G_{1}(\tau )=\frac{3P_{o}A\frac{{\mu_{s}}^{\prime }}{\mu_{\mathrm{tr}}}}{\left(\frac{\mu_{\mathrm{eff}}}{\mu_{\mathrm{tr}}}+1)(\frac{\mu_{\mathrm{eff}}}{\mu_{\mathrm{tr}}}+3A\right)}.$$ (3)

In Eq. (3), $P_{o}$ is the incident optical power, and A is a function of the tissue refractive index.^21^ All other terms in Eq. (3) are exclusively functions of the static and dynamic tissue absorption and scattering coefficients ($\mu_{a}$, ${\mu_{s}}^{\prime }$, $\mu_{a,\mathrm{dyn}}$). $\mu_{a,\mathrm{dyn}}$ is a function of $\left\langle r^{2}(\tau )\right\rangle$, and $\left\langle r^{2}(\tau )\right\rangle$ is related to the characteristic flow speed $v_{c}$ via the equation $\langle r^{2}(\tau )\rangle =v_{c}\tau^{2}$ (for directional flow).^24^ Using this framework and inputting the measured value of K from LSI and the measured $\mu_{a}$ and ${\mu_{s}}^{\prime }$ from SFDI at each time point, Eq. (1) was solved for $v_{c}$ at each time point and each pixel by iterating over a predefined grid of potential $v_{c}$ values and minimizing a least-squares cost function. The resulting spatiotemporal values of $v_{c}$ were used in place of SFI in the subsequent steps to achieve an optical property-corrected calculation of ${\mathrm{CMRO}}_{2}$.

#### 2.4.2 Absolute cerebral metabolic rate of oxygen calculation

To calculate absolute ${\mathrm{CMRO}}_{2}$, we start from the equation:^25^

$${\mathrm{CMRO}}_{2}=(\mathrm{CBF})(\mathrm{OEF})({\left[O_{2}\right]}_{a}).$$ (4)

In Eq. (4), CBF is the cerebral blood flow, ${\left[O_{2}\right]}_{a}$ is the arterial concentration of oxygen, and OEF is the oxygen extraction fraction, equal to $({\left[O_{2}\right]}_{a}-{\left[O_{2}\right]}_{v})/{\left[O_{2}\right]}_{a}$, where ${\left[O_{2}\right]}_{v}$ is the venous concentration of oxygen. For a single arteriole, $\left(\mathrm{OEF})({\left[O_{2}\right]}_{a}\right)$ represents the molar concentration of oxygen that was extracted from that arteriole and used by the brain for metabolic processes related to the synthesis of ATP. This quantity is equivalent to the molar concentration of deoxygenated hemoglobin that arrives in a nearby venule following oxygen extraction by the brain. Therefore, within our measurement paradigm,^11^ Eq. (4) is rewritten as

$${\mathrm{CMRO}}_{2}=4a(v_{c})({\mathrm{ctHb}}_{v})({\mathrm{Hb}}_{\mathrm{bl}}/{\left\langle {\mathrm{ctHb}}_{\mathrm{tot}}\right\rangle }_{p}).$$ (5)

In Eq. (5), ${\mathrm{ctHb}}_{v}$ is the tissue concentration of deoxygenated hemoglobin in an ROI atop a large vein in the ctHb maps obtained from SFDI. The factor of 4 accounts for the fact that the hemoglobin molecule has four binding sites for oxygen. Since $v_{c}$ is a characteristic flow parameter and not an absolute value of blood flow, it is necessary to include the proportionality constant α in the equation to convert $v_{c}$ into a quantity with units of absolute flow speed.

The factor (${\mathrm{Hb}}_{\mathrm{bl}}/{\left\langle {\mathrm{ctHb}}_{\mathrm{tot}}\right\rangle }_{p}$) accounts for partial-volume effects caused by the diffuse nature of light propagation in the brain. Equation (4) requires an intravascular oxygen concentration, but SFDI measures a bulk tissue deoxyhemoglobin concentration. Hence, a blood-volume fraction term is required to convert between these two quantities. The numerator, ${\mathrm{Hb}}_{\mathrm{bl}}$, is the concentration of hemoglobin in the blood sampled from the femoral artery of the animal during the arterial blood gas (ABG) measurement. The denominator, ${\left\langle {\mathrm{ctHb}}_{\mathrm{tot}}\right\rangle }_{p}$, is the mean total tissue hemoglobin concentration in the parenchyma during the period that the ABG was acquired. The factor (${\mathrm{Hb}}_{\mathrm{bl}}/{\left\langle {\mathrm{ctHb}}_{\mathrm{tot}}\right\rangle }_{p}$) enables the required conversion of our optical ctHb measurements from the scale of a tissue hemoglobin concentration to the scale of a vascular hemoglobin concentration, mitigating the partial volume effect and allowing us to measure ${\mathrm{CMRO}}_{2}$ on an absolute scale.

The parameter α is typically unknown; thus, the quantity reported in optical brain imaging studies is usually the relative ${\mathrm{CMRO}}_{2}$ (${\mathrm{rCMRO}}_{2}$). However, in this report, we were able to measure absolute ${\mathrm{CMRO}}_{2}$ using a “zero-flow” boundary condition,^26^ which is provided by the onset of global cerebral ischemia in our animal model:

$$4a(v_{c}){{|}_{T\mathrm{pre}}({\mathrm{ctHb}}_{v})|}_{T\mathrm{pre}}={4({\mathrm{dctHb}}_{v}/dt)|}_{T\text{post}}.$$ (6)

In Eq. (6), the vertical bars denote the time at which the variable was evaluated. This procedure was performed for each of the 10 subjects in this study, using the values of SFI and ${\mathrm{ctHb}}_{v}$ immediately before asphyxia ($T_{\mathrm{pre}}$) and the mean rate of change ${\mathrm{dctHb}}_{v}/\mathrm{dt}$ immediately after the onset of asphyxia ($T_{\text{post}}$). The value of ${\mathrm{dctHb}}_{v}/\mathrm{dt}$ was measured by fitting a sigmoid function to the ctHb curve during the beginning of the “zero-flow” period, finding the $t_{50}$ value of the sigmoid, linearizing the sigmoid within a 30-s window centered on the $t_{50}$ point, and calculating the slope of the resulting line segment. The values of α and absolute ${\mathrm{CMRO}}_{2}$ then were calculated over the entire craniectomy region at each measurement time point.

## 3 Results

**Fig. 2:**
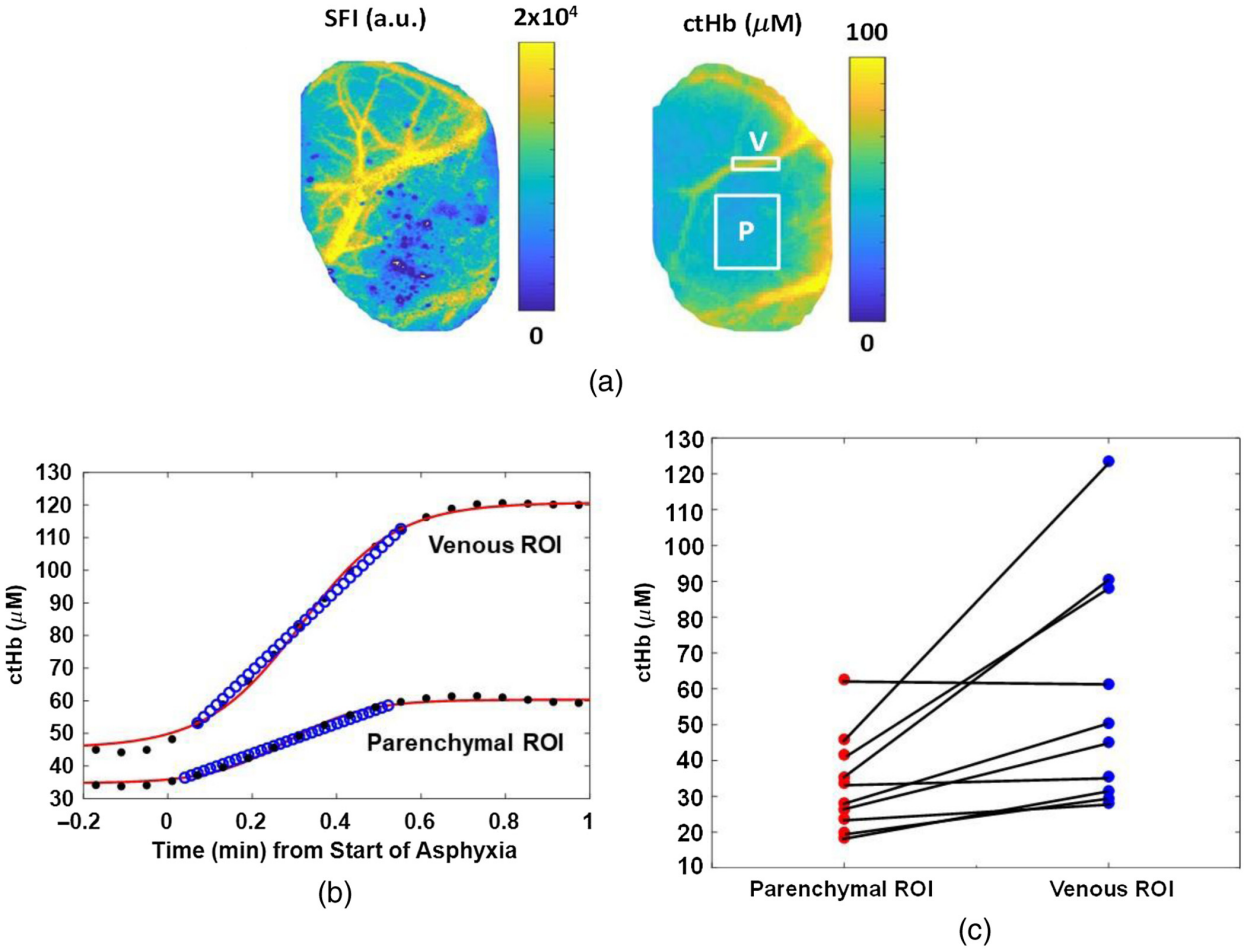
Venous and parenchymal ROIs demonstrate different hemodynamics in response to CA. (a) Images of blood flow (SFI) and deoxyhemoglobin concentration (ctHb), measured from the rat brain using LSI and SFDI, respectively. ROIs over the parenchyma (P) and large vein (V) are labeled in the ctHb image. (b) ctHb (black dots) increases in these ROIs during the initial minute of asphyxia. The sigmoidal fit (red line) and linear fit (blue circles) to the measured data are used to calculate the parameter α in Eq. (6). (c) The rate of change of tissue ctHb (dctHb/dt) during the initial minute of asphyxia is higher in the venous ROI than parenchyma ROI.

Immediately following CA, cerebral hemodynamics are spatially heterogeneous (Fig. 2). For example, the rate of change in cerebral ctHb following the start of asphyxia was 58.3±32.3  μM/min in an ROI selected over a large vein [Fig. 2(b)], but only 33.6±13.6  μM/min in an ROI selected over the parenchyma.

**Fig. 3:**
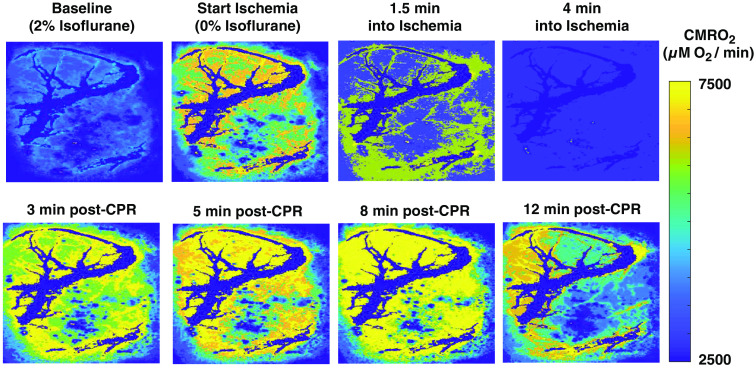
Absolute ${\mathrm{CMRO}}_{2}$ ($\mu M\;O_{2}/\min$) maps of a ∼6  mm×4  mm region of the rat brain at different time points during a CA/CPR experiment. Metabolic activity increases as anesthesia is being washed out (between “baseline” and “start ischemia”), followed by a sharp decrease during ischemia. Following CPR, ${\mathrm{CMRO}}_{2}$ recovers to anesthesia-free baseline level (3 min post-CPR), subsequently increases to values higher than baseline (5 to 8 min post-CPR), and then declines to values approaching anesthetized baseline level once cerebral electrical activity resumes (12 min post-CPR). Large vessels (dark blue) have been removed from the ${\mathrm{CMRO}}_{2}$ images to signify that the oxygen metabolism we are measuring is occurring in the parenchyma.

Maps of absolute ${\mathrm{CMRO}}_{2}$ throughout a representative CA/CPR experiment are shown in Fig. 3. At baseline, the animal is under anesthesia (2% isoflurane). After 2 min of anesthesia washout, the ${\mathrm{CMRO}}_{2}$ increased by a factor of ∼2 as the subject woke up. Following the onset of ischemia, the ${\mathrm{CMRO}}_{2}$ rapidly decreased as the subject entered CA. After resuscitation, the ${\mathrm{CMRO}}_{2}$ rapidly increased until reaching a maximum value at ∼8  min post CPR (during hyperemia). Subsequently, the ${\mathrm{CMRO}}_{2}$ decreased toward baseline as cerebral electrical activity resumed (∼12  min post-CPR).

**Fig. 4:**
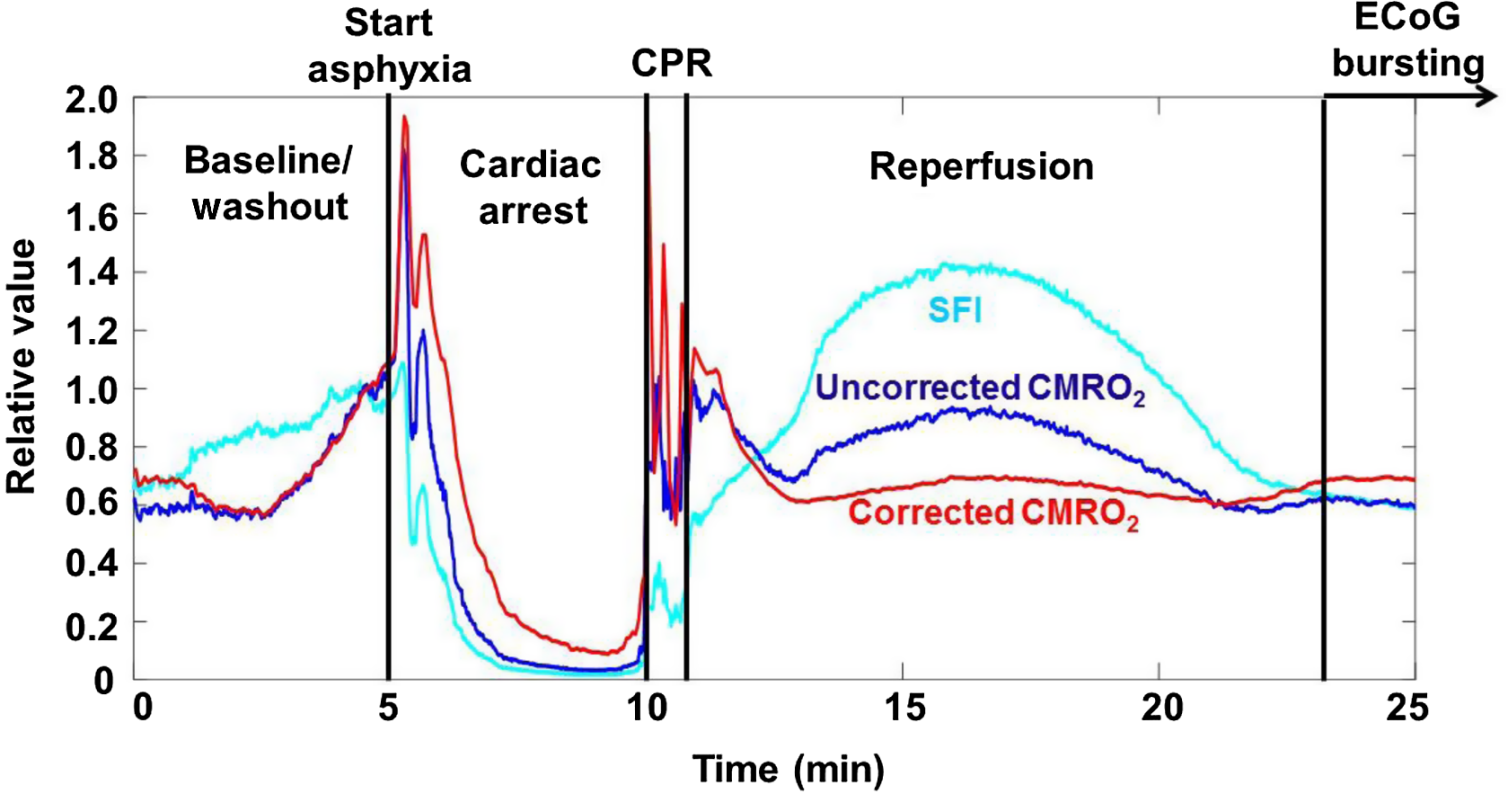
Optical properties affect calculation of ${\mathrm{CMRO}}_{2}.$ Comparison of SFI (light blue), ${\mathrm{CMRO}}_{2}$ calculated using SFI (uncorrected ${\mathrm{CMRO}}_{2}$, dark blue), and ${\mathrm{CMRO}}_{2}$ calculated using $v_{c}$ (corrected ${\mathrm{CMRO}}_{2}$, red), which accounts for the effects of tissue optical properties on SFI. For ease of comparison, the three curves are normalized to their value at a point near the end of the washout period (t∼4  min). This correction reveals differences in the observed rates of change in ${\mathrm{CMRO}}_{2}$ during reperfusion and resumption of ECoG bursting, suggesting the need to take optical properties into account even for relative ${\mathrm{CMRO}}_{2}$ measurements.

Calculation of changes in ${\mathrm{CMRO}}_{2}$ is affected by optical properties (Fig. 4). Optical properties measured with SFDI, along with Eq. (1), enable calculation of a characteristic flow speed ($v_{c}$) that can be used in place of SFI for the calculation of relative ${\mathrm{CMRO}}_{2}$ (${\mathrm{rCMRO}}_{2}$). During each of the experimental phases, a comparison of ${\mathrm{rCMRO}}_{2}$ trends suggests that the metabolic activity is at times greater (i.e., during the hyperemic phase) and lower (i.e., during CA) with use of SFI instead of $v_{c}$ in the calculation of ${\mathrm{CMRO}}_{2}$. This result demonstrates that changes in cerebral optical properties can affect calculations of ${\mathrm{CMRO}}_{2}$ dynamics using SFI alone to measure flow and hence illustrates the need to measure optical properties to properly characterize cerebral metabolic activity.

**Fig. 5:**
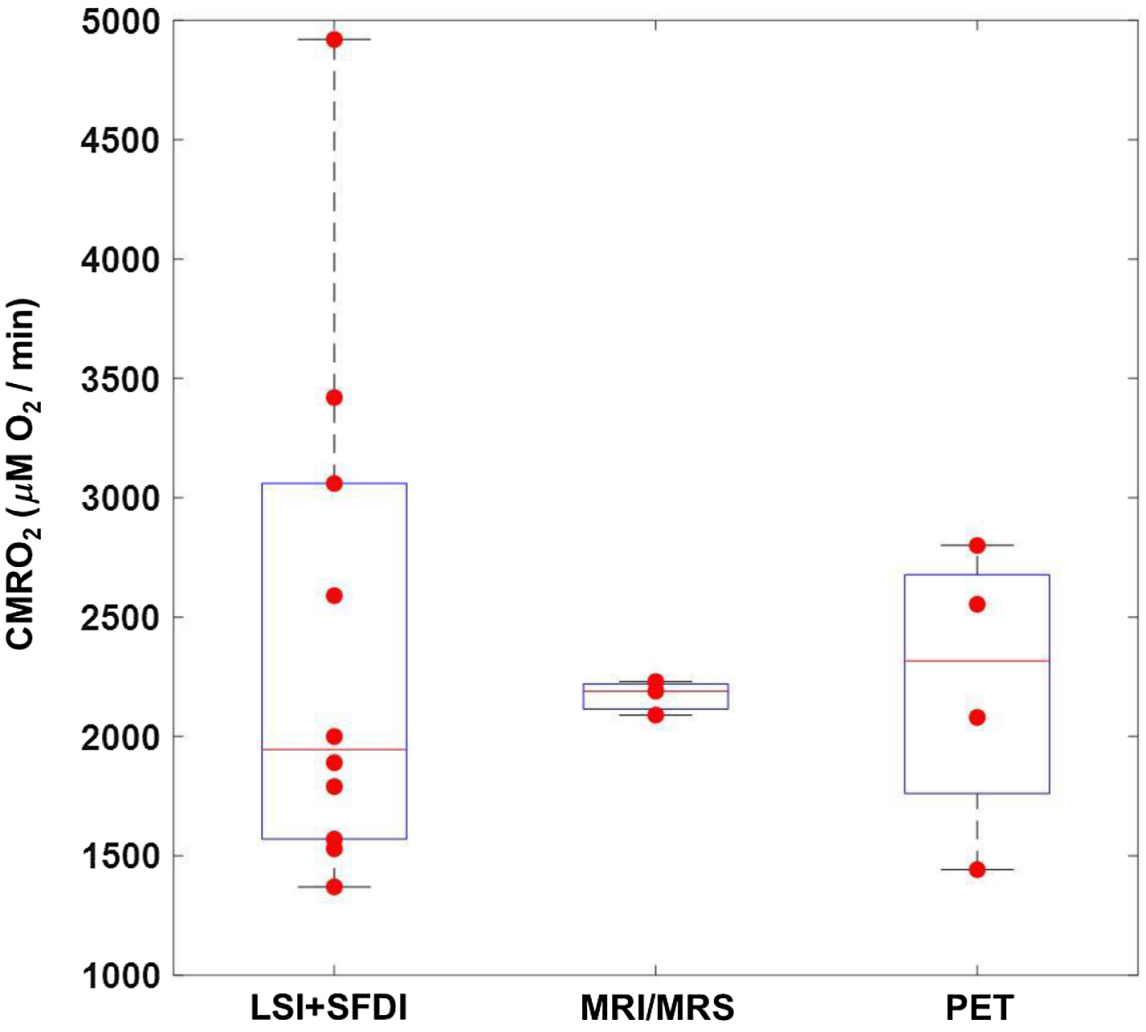
Absolute ${\mathrm{CMRO}}_{2}$ values ($\mu M\;O_{2}/\min$) measured with our combined SFDI + LSI optical system are in agreement with absolute ${\mathrm{CMRO}}_{2}$ values reported in preclinical studies using different imaging modalities (MRI/MRS, PET). Each LSI + SFDI data point represents an individual subject (n=10) imaged in this report. Each data point for MRI/MRS and PET represents the reported average ${\mathrm{CMRO}}_{2}$ of the subjects measured in individual studies.

Figure 5 shows distributions of ${\mathrm{CMRO}}_{2}$ values measured with our imaging setup as compared to values reported in the literature using various medical imaging approaches, including magnetic resonance methods (MRI/MRS)^27^^–^^29^ and PET.^3^^–^^7^ The LSI + SFDI method reported here measures absolute ${\mathrm{CMRO}}_{2}$ values that are within the range measured with these approaches, suggesting the accuracy of our optical imaging approach to determine ${\mathrm{CMRO}}_{2}$. It is important to note that in Fig. 5, each data point in the boxplot for our method represents an individual rat, but in the MRI/MRS and PET boxplots, each data point is the mean ${\mathrm{CMRO}}_{2}$ value over the group of rats used in each of the studies cited above. Therefore, it is not surprising that there is a greater spread in the ${\mathrm{CMRO}}_{2}$ values in Fig. 5 that were obtained from our method.

## 4 Discussion

Here, we provide, to the best of our knowledge, the first demonstration of dynamic imaging of absolute ${\mathrm{CMRO}}_{2}$ in the living brain using a combination of LSI and SFDI techniques. We use the tissue optical properties measured with SFDI to account for their effects on interpretation of the LSI information. We then use the “zero-flow” condition inherent in our CA experimental paradigm to solve for the coefficient α in the ${\mathrm{CMRO}}_{2}$ equation using a continuity condition at the boundary between normal flow and zero-flow states. Using this technique, we perform quantitative spatial mapping of absolute ${\mathrm{CMRO}}_{2}$ continuously throughout the different stages of the CA + CPR experiment. The ${\mathrm{CMRO}}_{2}$ obtained from our optical system agreed well with established brain imaging techniques (PET, MRI/MRS).

This paradigm for measuring absolute ${\mathrm{CMRO}}_{2}$, in units of $\mu M\;O_{2}/\min$, enables direct comparison of metabolic activity among subjects, across separate imaging sessions, and on different days for a single subject. This approach potentially enables longitudinal monitoring of cerebral recovery for days or weeks following ischemia and reperfusion. The methods described here can be applied to quantitative measurement of metabolic recovery and flow-metabolic coupling and uncoupling in preclinical models of ischemic conditions such as CA and stroke.

It is important to note that in this study, we observed large variation among the absolute ${\mathrm{CMRO}}_{2}$ values for the individual animals; this may be attributed to the lengthy experimental procedures performed prior to the measurements. For these animals, several hours of surgery took place on the day of the experiment to implant electrophysiology leads, intubate and cannulate the animals, and perform the partial craniectomy for optical imaging. These procedures likely contributed to stress in the animals that could have resulted in significant variation in cerebral metabolic state between the animals at the time the measurements were performed. To address this issue, future studies can reduce stress on the animals by (1) employing a thinned-skull technique instead of a craniectomy and (2) implanting electrophysiology leads on a day prior to the experiment.

### 4.1 Optical Imaging Segments Venous Regions to Better Quantify Cerebral Oxygen Extraction

The imaging capability of our device allows the segmentation of an ROI atop a prominent vein, which enables more accurate measurements of the quantity of deoxygenated venous blood and, hence, the quantity of oxygen consumed by the brain. With the use of a larger ROI, the local ${\mathrm{CMRO}}_{2}$ would be systematically underestimated due to inclusion of the parenchyma in the ROI, as oxygen extraction in the parenchyma is lower than in individual vessels. ${\mathrm{CMRO}}_{2}$ models of diffuse light transport implicitly assume that the concentration of deoxygenated hemoglobin is that within the veins specifically and not the bulk tissue.^30^ However, most diffuse optics measurements of ${\mathrm{CMRO}}_{2}$ are unable to satisfy this condition, as they typically use fiber-based spectroscopic techniques that sample the bulk tissue and thus cannot distinguish between venous and mixed arterial-venous parenchymal regions. In this report, the use of DOI allows us to obtain a spatial map of the tissue properties, enabling use of deoxyhemoglobin concentrations measured in a venous ROI to obtain more accurate quantitative values of ${\mathrm{CMRO}}_{2}$.

### 4.2 Correction of ${\mathrm{CMRO}}_{2}$ Data for Partial-Volume Effects

Due to the heterogeneity of biological tissues, partial-volume effects are a well-known confounding factor, especially with optical property mapping using a planar wide-field imaging technique such as SFDI.^19^ In our work, we require knowledge of ${\mathrm{Hb}}_{v}$ [Eq. (6)]. However, due to partial volume effects, simple selection of an ROI that is coincident with a venule is insufficient. To address this issue, we employed a partial-volume correction to the ${\mathrm{CMRO}}_{2}$ equation. To accurately incorporate this scaling term, it is necessary to know the concentration of total hemoglobin (${\mathrm{Hb}}_{\mathrm{bl}}$) within the blood of each animal. In this report, these values were acquired via ABG measurement before CA. A coefficient of variation of 13% in ${\mathrm{Hb}}_{\mathrm{bl}}$ was determined from the measurements. If the variation in ${\mathrm{Hb}}_{\mathrm{bl}}$ among the different subjects was not considered, an additional error of ∼12% to 25% in the measured ${\mathrm{CMRO}}_{2}$ would be achieved due to this within-group variability in ${\mathrm{Hb}}_{\mathrm{bl}}$ values.

### 4.3 Contributions of Directed Flow Versus Diffuse Flow

With diffuse optical measurements of blood flow, the model of blood flow typically is assumed to be either diffusion-like (i.e., Brownian motion) or directional (i.e., intravascular).^31^ Here, we assumed that the corrected flow speed was the latter [Eq. (2)]. Previous studies have used the diffusion-like term as the free parameter when fitting for flow speed^32^ or constrained the fit in a model system such that one could choose to fit for either diffuse or directed flow but not both simultaneously.^24^ Recently, Postnov et al.^33^ used high-speed LSI to map the autocorrelation function pixel-by-pixel in the rodent brain, identifying the dominant type of particle motion at each pixel. They observed that the directed flow term was dominant in large vessels, whereas the diffuse flow term was dominant in the parenchyma.

Here, we could not rigorously solve for the autocorrelation function because the sampling frequency of our LSI data acquisition was too low to perform a method similar to that of Postnov et al.^33^ Instead, we used a two-step approach of (1) using SFDI data to account for the effects of optical properties on interpreting the LSI data and (2) fitting the resulting corrected data to a model of directed flow to extract a characteristic flow speed. This method provided characteristic flow speeds that were similar to previously reported values.^34^

### 4.4 Limitations of Zero-Flow Condition

Our current approach for measuring absolute ${\mathrm{CMRO}}_{2}$ requires temporary induction of a “zero-flow” condition in the brain. In this report, this condition was met using a CA model in rats. However, there is a clear need to investigate alternative approaches for interrogating absolute ${\mathrm{CMRO}}_{2}$ without creating harmful perturbations. Future work can incorporate techniques such as balloon occlusion tests, as sometimes done in the clinical setting,^35^ to temporarily induce a zero-flow condition that can be quickly reversed without long-term harm to the animal. However, these procedures may have longer-term effects on the brain, so they would require evaluation within our preclinical model. Although the comparison of absolute ${\mathrm{CMRO}}_{2}$ calculated with our approach with PET and MRI is encouraging, further comparison work is required with measurements collected from the same animals under identical anesthesia conditions. Future work in this area is warranted. Additional future studies will assess the sensitivity of our ${\mathrm{CMRO}}_{2}$ method to small perturbations in brain metabolism over time in longitudinal studies. This future investigation will involve comparison of our method with an established modality such as MRI in preclinical chronic imaging experiments.

## 5 Conclusion

Here, we have described, to the best of our knowledge, the first report of absolute ${\mathrm{CMRO}}_{2}$ mapping in the rat brain using DOI. Absolute ${\mathrm{CMRO}}_{2}$ allows for quantitative assessment of cerebral metabolism without the need for baseline measurements, enabling longitudinal comparison between animals and among multiple days of measurement on an absolute rather than relative scale. The ${\mathrm{CMRO}}_{2}$ measurements provided by our multimodal system were in good agreement with those previously measured in the brain of anesthetized rats using PET and MRI. This method shows significant potential for assessing and monitoring cerebral metabolism and predicting cerebral response to ischemic injury.

## Code, Data, and Materials Availability

Custom MATLAB code used in this report is available upon request by contacting Prof. Robert Wilson (wilsonrh@uci.edu).
